# Thyroid status in pregnancy: Comparison of thyroid function abnormalities in women with and without a history of miscarriage or stillbirth

**DOI:** 10.12669/pjms.40.1.7282

**Published:** 2024

**Authors:** Ghulam Abbas, Samina Aliya Sabir, Siddiq Ur Rehman, Beenish Gohar

**Affiliations:** 1 Ghulam Abbas, FCPS Department of Medicine, Khyber Teaching Hospital Peshawar, Pakistan; 2 Samina Aliya Sabir, FCPS Department of Obstetrics & Gynecology Lady Reading Hospital, Peshawar, Pakistan; 3 Siddiq Ur Rehman, MBBS, MRCP Royal Liverpool University Hospital, UK; 4 Beenish Gohar, MBBS, Department of Obstetrics & Gynecology Hayatabad Medical Complex, Peshawar, Pakistan

**Keywords:** Hypothyroidism, Hyperthyroidism, Miscarriage, Pregnancy, Stillbirth, Sub clinical hypothyroidism

## Abstract

**Objective:**

To evaluate thyroid function tests (TFTs) during pregnancy in women with previous history of miscarriage or stillbirth.

**Methods:**

A cross-sectional study was carried out at the department of Obstetrics & Gynaecology and Endocrinology, Lady Reading Hospital, Peshawar from February 2021 to March 2022. All multigravida women attending the antenatal clinics were included using consecutive sampling. These women were placed into two groups, Group-A comprised of women with no prior history of miscarriages or stillbirths, and those with a history of foetal death during previous pregnancies were assigned Group-B. Free T4, thyroid stimulating hormone (TSH) and anti-thyroid peroxidase (TPO) antibodies were measured and the former two were used to label patients with thyroid dysfunction.

**Results:**

A total of 139 multigravida women were included in the study. About 43% of the women had a history of miscarriages or stillbirths. Thyroid dysfunction was observed overall in 36.69 % women, of whom 25.18% had sub-clinical hypothyroidism, 6.47% had hypothyroidism and 5.04 % were sub-clinical hyperthyroid. Women in Group-B had more thyroid functions abnormalities compared to Group-A (p<0.05). Moreover, there was significant difference in median TSH and freeT4 between the groups (p<0.001). Overall, thyroid dysfunction was found in 66.67% of patients who had a history of foetal death.

**Conclusions:**

In pregnant women with a history of miscarriage or abortion, thyroid functions abnormalities are common therefore routine thyroid testing is advised in pregnant women to prevent adverse perinatal outcomes.

## INTRODUCTION

Thyroid disease is common in women of reproductive age, and globally, after diabetes, it is the most prevalent endocrine disorder at this age.^1^ The impact of maternal thyroid function on reproductive health and pregnancy outcomes has been investigated in a number of studies during the past decade. It is estimated that 2%-3% of pregnancies are affected by thyroid abnormalities. Hyperthyroidism affects in 0.2%-0.4% of pregnant women, with Graves’ disease being the most common cause of it.^2^-^4^ The incidence of hypothyroidism is between 2-3%, overt hypothyroidism affects between 0.2% and 1% of all pregnancies. The prevalence of subclinical hypothyroidism (SCH) varies with ethnicity, iodine intake, and the definition used and is reported to range between 2% and 3%.^3^,^4^

Thyroid abnormalities are reported to be linked with adverse pregnancy outcomes.^4^,^5^ Hypothyroidism as well as overt and SCH are associated with high probabilities of eclampsia, pre-eclampsia, gestational hypertension, anaemia, placental abruption, low-birth-weight (LBW), cognitive dysfunction, and increased perinatal mortality.^6^ With hyperthyroidism during pregnancy, complications such as still-birth, abortion, premature birth, pre-eclampsia, heart failure, and thyroid storm may develop. Similarly, uncontrolled thyrotoxicosis can lead to pre-eclampsia, intrauterine growth retardation, LBW, and miscarriages.^3^,^4^ Literature have reported mixed results and the actual foetal loss has not been well described.^7^

Some studies have reported fourfold higher risk of poor foetal outcome when TSH level is elevated.^7^,^8^ Similarly perinatal mortality has been reported for thyroid peroxidase antibodies(TPO) positive mothers.^9^ Some other published studies observed no association between thyroid abnormalities and perinatal death.^7^,^10^ As thyroid abnormality is thought to be linked with adverse pregnancy outcomes, hence, thyroid testing is frequently advised as part of antenatal tests to evaluate adverse perinatal outcomes. Furthermore, it is not clear to what level periodic thyroid testing in women who have experienced a still-birth contributes to proportions of women with thyroid dysfunction that are higher than those observed in a comparable population without still-birth. Considering this, the present study was conducted to evaluate thyroid function testing during pregnancy in women who had a previous history of miscarriage or stillbirth.

## METHODS

This cross-sectional study was carried out on pregnant women presented to the department of Gynaecology and Endocrinology, Lady Reading Hospital, Peshawar from February 2021 to March 2022. Sample size was computed taking earlier reported 10% thyroid abnormalities in pregnancy.11 A total of 139 patients were included using consecutive sampling.

### Inclusion & Exclusion Criteria

Multigravida Pakistani women were included while primigravida and all other pregnant women with known thyroid or metabolic abnormalities before pregnancy or on thyroid medications were excluded. In addition, women with any chronic or acute severe illness and those with hyperemesis gravidarum and multiple pregnancies were also excluded. Serum levels of free T4 and TSH in the general population typically range from 0.89 to 1.76 ng/dL and 0.35 to 4.5 mU/L, respectively. . We classified different groups of thyroid status using trimester-specific values of TSH as per the American Thyroid Association (ATA) guidelines. According to these guidelines, TSH values should be 0.1–2.5 mIU/L during the first trimester, 0.2–3.0 mIU/L during the second trimester, and 0.3–3.5 mIU/L during the third trimester^2^

A concentration of >50IU/ml was considered positive for anti-TPO antibodies. These women were divided into two groups, Group-A comprised of women with no prior history of miscarriages or stillbirths, and those with a history of foetal death during previous pregnancies were assigned Group-B. Death of foetus occurred <22 weeks of gestation was termed as miscarriage while stillbirth was defined as death occurred from 22 weeks of gestation until delivery.^12^

### Ethical Approval

Ethical approval was taken from the institution’s ethical review committee (Reference No: 32/LRH/MTI; dated: 08/02/2021).

Mean (SD) or median (IQR) were calculated for variables with continuous nature while categorical variables were expressed as frequency and percentage. Association between categorical variables was determined using chi-square test or Fisher’s exact test where applicable. The differences were calculated using student independent t-test or Mann Whitney for variables with two categories and one-way ANOVA/ Kruskal Wallis test was used for more than two categories where appropriate. Spearman rank correlation was used to look into correlation. The significance level was set at p<0.05 for statistical tests. SPSS version 21® was used for analysis.

## RESULTS

**Fig.1:**
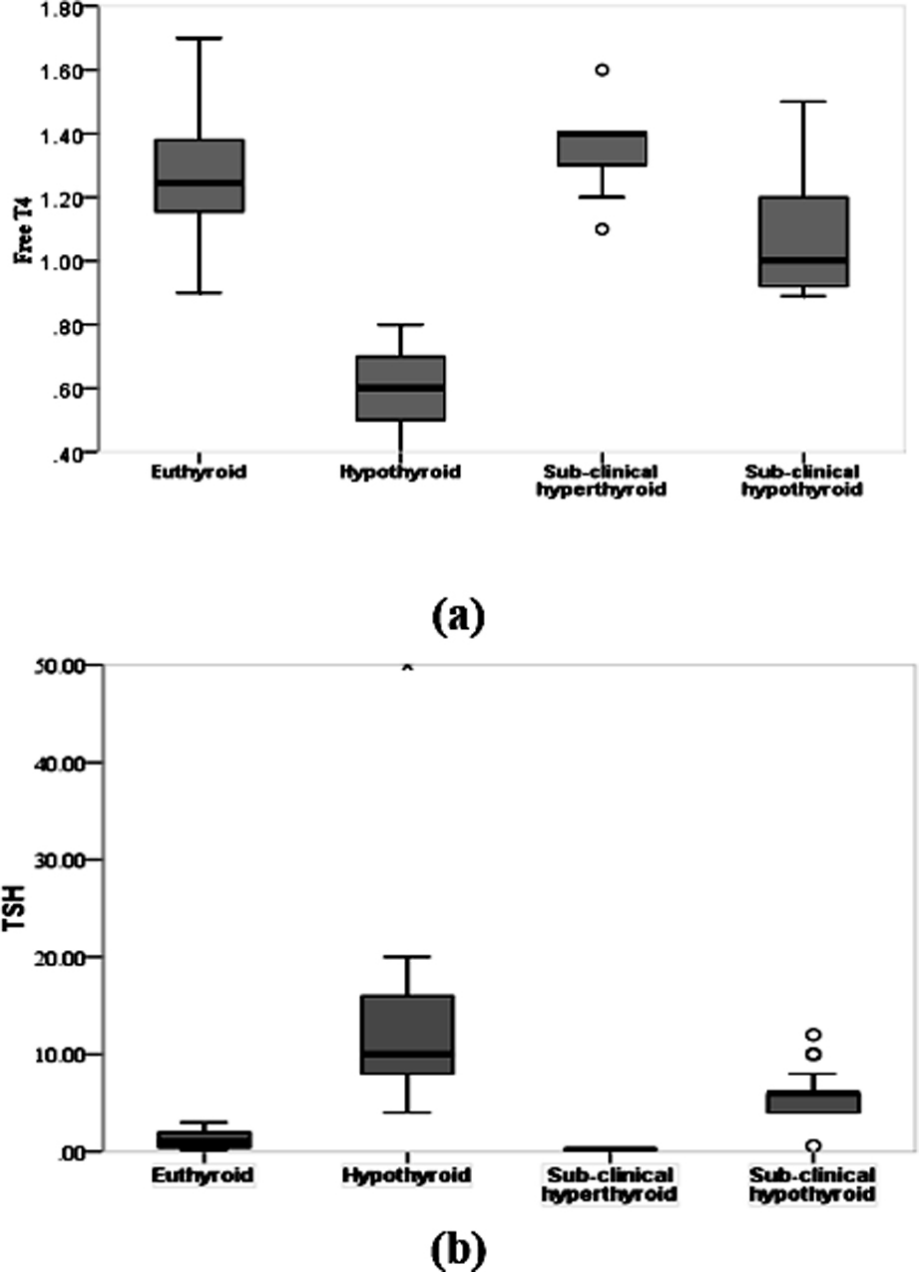
Post-hoc pairwise median comparisons of TSH (a) and Free T4 (b).

The maternal mean age was 30.05±5.23 years. Obstetric characteristics showed that mean of gravida and parity was 3.57±1.19 and 2.01±1.03 respectively. About 43% (n=60) of the women had a history of miscarriages or stillbirths. Serum TSH and FT4 of the study participants was 2.00(3.60) mIU/ml and 1.16±0.24 pmol/L respectively. Anti-TPO antibodies test was conducted for a total of 38 patients where positive for anti-TPO antibodies presence was observed in 94.7% (n=36) patients. Normal thyroid function was observed in over 63.0% of the women. All the patients with thyroid dysfunction 36.69 % (n=51) were further categorized based on the function. Of the total, 25.18% (n=35) had SC followed by hypothyroidism 6.47% (n=9) and sub-clinical hyperthyroid 5.04% (n=7). None of the patients was found to be hyperthyroid. In group of hypothyroid patients, the majority 44.44% was observed during the second trimester of pregnancy. Similarly, among those with sub-clinical hyperthyroid an equal percentage of 42.86% was observed during both the second and third trimesters. Notably, sub-clinical hypothyroidism was detected in 37.14% of the patients specifically during the third trimester. Maternal age was higher in SCH group as compared to hypothyroid, euthyroid and sub-clinical hyperthyroid (p=0.077). Women with hypothyroidism had a mean gravidity 4.44±1.59 and the differences were significantly different across thyroid dysfunction (p=0.003). Similarly median differences of serum TSH and FT4 were statistically significantly different between the groups. Pairwise analysis was conducted to see within the group differences (Fig.1). Within the group of SCH, 22 patients were screened for anti-TPO antibodies and all of them were positive. A similar pattern was observed for hypothyroid (Table-I).

**Table-I:** Comparison of characteristics with euthyroid women with those with thyroid abnormalities.

Characteristics		Euthyroid N=88	Hypothyroid N=9	Sub-clinical hyperthyroid N=7	Sub-clinical hypothyroid N=35	p-value
Maternal age(years) Mean± SD		29.34±5.30	31.67±6.67	28.43±4.69	31.74±4.41	0.077
Gravidity, Mean± SD		3.34±1.13	4.44±1.59	3.14±0.90	4.00±1.06	0.003
Parity, Mean± SD		2.06±1.08	2.11±1.05	1.86±0.69	1.89±0.96	0.819
Gestational age, n (%)	First trimester	14(15.91)	2(22.22)	1(14.29)	11(31.43)	0.561
	Second trimester	38(43.18)	4(44.44)	3(42.86)	11(31.43)	
	Third trimester	36(40.91)	3(33.33)	3(42.86)	13(37.14)	
Serum TSH, Median (IQR)		1.00(1.60)	10.00(12.00)	0.20(0.10)	6.00(2.00)	<0.001
Serum FT4, Median (IQR)		1.25(0.24)	0.60(0.25)	1.40(0.20)	1.00(0.30)	<0.001
Anti-TPO antibodies, n (%)	Positive	9(81.82)	5(100.00)	0(0.00)	22(100.00)	0.075
	Negative	2(18.18)	0(0.00)	00.00)	0(0.00)	

Spearman rank correlation was used to see a relationship between TSH and FT4 for the entire sample highly statistically significant negative correlation was observed (r_s=_-0.546, p=0.01). Those screened for the presence of anti-TPO antibodies, positive patients had higher values of TSH and normal FT4 compared to the negative (p>0.05). In total, there were 43.20% (n=60) foetal deaths (Table-II). Age was significantly higher in Group-B (Patients with a history of foetal death during previous pregnancies) compared to Group-A (no prior history of miscarriages or stillbirths), and the difference was statistically significant.

**Table-II:** Comparison of maternal characteristics, TSH, T4 and thyroid abnormalities as risk factors for foetal loss.

Characteristics		Group-A (No-foetal death) N= 79	Group-B (Foetal death) N=60	p-value
Maternal age(years) Mean± SD		29.14±5.23	31.25±5.03	0.018
Gravidity, Mean± SD		3.11±0.95	4.17±1.21	<0.001
Parity, Mean± SD		2.11±0.95	1.87±1.11	0.160
Gestational age, n (%)	First	13(16.46)	15(25.00)	0.408
	Second	32(40.51)	24(40.00)	
	Third	34(43.04)	21(35.00)	
Hypothyroidism, n (%)	Yes	1(1.27)	8(13.33)	0.005
	No	78(98.73)	52(86.67)	
Sub-clinical hypothyroidism, n (%)	Yes	5(6.33)	30(50.00)	<0.001
	No	74(93.67)	30(50.00)	
Sub-clinical hyperthyroidism, n (%)	Yes	5(6.33)	2(3.33)	0.699
	No	74(93.67)	58(96.67)	
Serum TSH, Median(IQR)		1.00(1.60)	4.50(4.00)	0.001
Serum FreeT4, Median(IQR)		1.24(0.24)	1.00(0.30)	<0.001
Anti-TPO antibodies, n (%)	Positive	0(0.00)	36(94.74)	NC
	Negative	0(0.00)	2(5.26)	
Thyroid dysfunction, n (%)	Present	11(13.92)	40(66.67)	<0.001
	Absent	68(86.08)	20(33.33)	

NC: not computable.

In order to reduce statistical bias, thyroid dysfunction was individually cross tabulated with groups. Hypothyroidism was present in 1.27% (n=1) of the women in Group-A and 13.33% (n=8) in Group-B, and there was a statistically significant association between them (p= 0.005). SCH was observed in 6.33% (n=5) in Group-A, and 48.33% (n=29) in Group-B, and the difference in the proportion was significant (p<0.001). Sub-clinical hyperthyroidism was found in 6.33% (n=5) and 3.33% (n=2) in groups A and B respectively. The median TSH level was 4.50(4.00) in Group-B and 1.00(1.60) in Group-A, and the difference was statistically significant (P=0.001). Similarly, median serum FT4 was significantly different between Group-A and B (1.24 vs 1.00, P<0.001). As a whole thyroid dysfunction was found in 13.92 % (n=11) of Group-A and 66.67% (n=40) of Group-B. Anti-TPO antibodies were positive in 94.74% (n=36) and negative in 5.25%) (n=2) in Group-B. In patients with thyroid dysfunction, anti-TPO antibodies were positive in 100% (n=27) patients and negative in 81.80% (n= 9) patients (p= 0.078).

## DISCUSSION

In this study, 43% of the women had experienced miscarriages or stillbirths. Thyroid dysfunction was prevalent among 36.69% of the women, including 25.18% with SCH, 6.47% with hypothyroidism, and 5.04% with sub-clinical hyperthyroidism. Those with a prior history of miscarriages or stillbirths had more thyroid function abnormalities. Among the women with miscarriage histories, Anti-TPO antibodies were assessed in 38 patients. Importantly, these antibodies were more prevalent among women with thyroid dysfunction compared to those without such dysfunction.

Hypothyroidism reported in this study is similar to the results reported by local study.^13^ There is wide variation in the prevalence of hypothyroidism in pregnancy, which is more common in Asians compared to Westerns.^11^,^14^ In Indian women the prevalence has been reported to range from 4.8% to 13%, while studies from the Middle East reported prevalence as high as 21%.^11^,^14^-^16^ Some characteristics of hypothyroidism are difficult to detect because it overlap with the sign of pregnancy itself. Furthermore, it varies with geographical area because of the iodine quantity in common salt and its consumption. SCH was the commonest in the study patients. Its prevalence ranges from 15% to 28% in different regions. A prevalence as high as 65% has been recorded, while other studies show a low frequency of just <2%.^13^,^17^,^18^ Western studies reported low frequency as compared to studies conducted in this part of the world. It may be because of the high prevalence of iodine deficiency in our region.^14^

Hyperthyroidism and subclinical hyperthyroidism are uncommon in pregnancy. Their prevalence ranges from 0.2 % to 1%. In this study, subclinical hyperthyroidism was found in 5% of women, while there were no cases of hyperthyroidism. Sub-clinical hyperthyroidism in the general population ranges from 0.6 to 16%. Our results are consistent with the earlier published studies. The most common cause of maternal hyperthyroidism is an autoimmune disorder. Women with hypothyroidism can have an increased risk of abortions and other pregnancy complications.^7^,^13^ Our study showed a strong association between the presence of hypothyroidism and abortions or miscarriages. Hypothyroidism was found in 13.33% of those with a previous history of abortions, as compared to 1.27% without abortions. A similar association has also been found in earlier studies.^7^,^19^ A meta-analysis by Zhang Y et al. revealed similar results.20 The association between SCH and obstetric complications is well established.^7^,^21^,^22^

In our study SCH was found in 48.33% of women having previous abortions in contrast to just 6.33% of those without abortions. Similar studies have also proven a strong association between maternal SCH and adverse foetal and pregnancy outcomes.^7^,^23^ Placental hypoplasia and other placental defects appear to be more frequently the cause of foetal loss in hypothyroid women. However, it is challenging to establish a link between placental hypoplasia and hypothyroid condition because of the combined placental abnormalities caused on by the various patterns in each patient. Additionally, there may be a correlation between foetal hydrops and sub-clinical hyperthyroidism. Although hyperthyroidism is associated with poor pregnancy outcomes, the effect of sub-clinical hyperthyroidism is controversial. Our study did not reveal an association between sub-clinical hyperthyroidism and a history of poor pregnancy outcomes. Our findings are supported by the study conducted by Casey et al.^24^

Anti-TPO antibodies were only measured in 38 women with a history of miscarriages. These antibodies were more common in women with thyroid dysfunction than without thyroid dysfunction which is in line with study conducted by Jantikar et al.^25^ The presences of thyroid dysfunctions were strongly associated with a history of abortions and miscarriages in this study. Our results are supported by many local and international studies.^7^,^8^,^10^,^13^,^24^,^26^ A study conducted by Shariatzadeh et al has found a strong association between maternal thyroid dysfunction and abortions or miscarriages in Iran.^27^ Thyroid dysfunctions are not only associated with abortions but are also responsible for several worse foetal and maternal outcomes. In this study raised serum TSH levels were significantly associated with the history of miscarriages. Such association has been observed and reported in literature published earlier.^7^,^28^ We found a significant difference between the levels of T4 in the two groups. which points towards hypothyroidism which is strongly associated with premature foetal loss.^29^

### Limitations

It includes small sample size. Prospective studies would be better to find more about the association between thyroid function abnormalities and miscarriages. Due to the poor socioeconomic conditions of the majority of our patients, we could not do all investigations on every patient.

## CONCLUSION

In pregnant women with a history of miscarriage or abortion, thyroid functions abnormalities are common therefore routine thyroid testing is advised in pregnant women to prevent adverse perinatal outcomes. It is therefore pertinent to screen women for thyroid dysfunction during each trimester of pregnancy to prevent possible complications. However, prospective studies are needed to establish a strong association to prove the benefit of managing thyroid abnormalities in these women.

### Author’s contributions:

**GA & SAS:** Conception, design & data collection.

**GA, SUR & BG:** Data analysis and interpretation/results.

**GA, SUR & BG:** Manuscript drafting and writing.

**GA & SUR:** Language editing, critical revision.

All authors read and approved the paper. The principal investigator is responsible and accountable for the accuracy or integrity of the work.
